# Ensuring a safe(r) harbor: Excising personally identifiable information from structured electronic health record data

**DOI:** 10.1017/cts.2021.880

**Published:** 2021-12-09

**Authors:** Emily R. Pfaff, Melissa A. Haendel, Kristin Kostka, Adam Lee, Emily Niehaus, Matvey B. Palchuk, Kellie Walters, Christopher G. Chute

**Affiliations:** 1 Department of Medicine, UNC Chapel Hill School of Medicine, Chapel Hill, North Carolina, USA; 2 University of Colorado Anschutz Medical Campus, Aurora, Colorado, USA; 3 The OHDSI Center at the Roux Institute, Northeastern University, Portland, Maine, USA; 4 TraCS Institute, University of North Carolina at Chapel Hill, Chapel Hill, North Carolina, USA; 5 Palantir Technologies, Denver, Colorado, USA; 6 TriNetX LLC, Cambridge, Massachusetts, USA; 7 Schools of Medicine, Public Health, and Nursing, Johns Hopkins University, Baltimore, Maryland, USA

**Keywords:** Electronic health records, data privacy, medical terminologies

## Abstract

Recent findings have shown that the continued expansion of the scope and scale of data collected in electronic health records are making the protection of personally identifiable information (PII) more challenging and may inadvertently put our institutions and patients at risk if not addressed. As clinical terminologies expand to include new terms that may capture PII (e.g., Patient First Name, Patient Phone Number), institutions may start using them in clinical data capture (and in some cases, they already have). Once in use, PII-containing values associated with these terms may find their way into laboratory or observation data tables via extract-transform-load jobs intended to process structured data, putting institutions at risk of unintended disclosure. Here we aim to inform the informatics community of these findings, as well as put out a call to action for remediation by the community.

## Introduction

Responsible clinical informatics professionals spend an enormous amount of time thinking about the Health Insurance Portability and Accountability Act (HIPAA) [1]. We do so not only to comply with legal requirements but also to protect patient privacy and engender public trust in our work. Hundreds of millions of patients across the US entrust health systems with their electronic health record (EHR) data; as informaticians, our end of that bargain is to use those data to advance the science of medicine and improve health and outcomes for those same “data donors.” Among other critical data protections, our continued ability to do this work depends on our capacity to guarantee the security or removal of the 18 identifiers recognized by HIPAA as personally identifiable information (PII) in the data we use [2]. However, our recent findings have shown that the continued expansion of the scope and scale of data collected in EHRs are making the protection of PII more challenging and may inadvertently put our institutions and patients at risk if not addressed. Here we aim to inform the informatics community of these findings, as well as put out a call to action for remediation by the community.

Protection or removal of PII from EHR data first requires inventorying the data fields in which PII can appear. This task has always been challenging within unstructured EHR data, such as free-text clinical notes − identifiers can appear anywhere within such text and are difficult to remove reliably without specialized software tools [3]. Removing PII from structured data (e.g., patient demographics, diagnoses, laboratory results) is a seemingly more straightforward task; fields that may contain PII are generally easily identified (“PATIENT_NAME,” “ADMISSION_DATE,” etc.) and can be dropped or masked in an automated fashion. Generally, research data warehouses are populated via extract-transform-load (ETL) processes directly from raw EHR data or an upstream enterprise data warehouse. These ETL processes can be built to remove some or all structured PII fields before data even reaches users.

## Problem Statement

During a recent quality audit of the research data warehouse at one of our institutions, it was discovered that unexpected records were appearing in the structured lab data. These records were coded with Logical Observation Identifiers Names and Codes (LOINC), the common standard for codifying laboratory data, and came in through the laboratory data ETL process − but these records were not laboratory data, but rather patient identifiers. This is because the complete LOINC vocabulary has a much wider scope than laboratory data and in fact can be used to code many other types of clinical observations and measurements. A list of the codes discovered in this initial audit is shown in Table 1 − notably, all of these codes fall into LOINC’s “Clinical” (not “Laboratory”) division.

**Table 1.** Example identifier-containing Logical Observation Identifiers Names and Codes (LOINC) codes

Code	Description
42077-8	Patient home phone
45401-7	Patient zip code
56799-0	Patient street address
68997-6	Patient city
87721-7	Patient county

This discovery was surprising and unnerving; not only were we unaware that PII could be mixed in among structured laboratory data but we also were not immediately able to assess the true scope of the problem. We had identified a handful of codes, but it was unknown how many more codes with PII attached had not yet been identified. This finding had broad implications; essentially, any past data extraction that included an unfiltered “dump” of laboratory or observation data had the potential to contain these codes, even if those extractions were intended to be deidentified. Once we fully investigated, engaged our privacy office, and remediated the issue, we felt it necessary to publicize these findings.

**Fig. 1.**
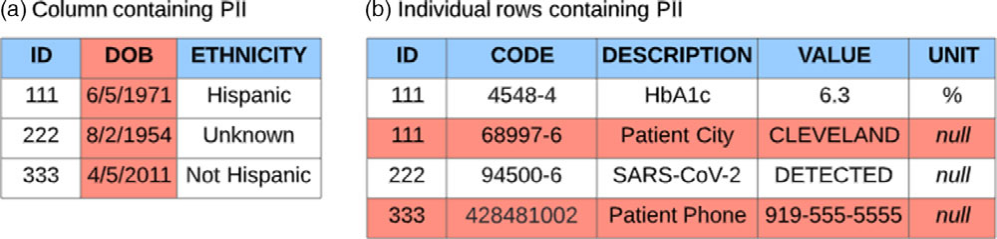
Removing an entire column known to contain personally identifiable information (PII) (a) is significantly easier than identifying PII-containing rows (b) that exist among nonidentifying records.

Broadly, structured EHR elements can be categorized as either self-contained, discrete facts (“the patient’s birthdate is **9/4/1998**”) or as code-value pairs (“the patient had an HbA1c test [**LOINC: 4548-4**], and the result of that test was **6.3%**”). Another way to mentally frame this common data structure is as “questions” (“What is the patient’s A1c?”)_and “answers” (“The patient’s A1c is 6.3%”). Discrete facts are much easier to assess for the presence of PII than code-value (question-answer) pairs. When dealing with a birthdate column, one can be reasonably assured that (1) all of the values in that column will be dates, presumably birth dates, and thus (2) all values in that column should be considered PII. Code-value pairs are much more variable and harder to handle in a consistent way. The “value” part of the pair is where PII may occur − however, the vast majority of data in the value column is not identifying (like our HbA1c result) and therefore would not ordinarily be suppressed. Removing PII from code-value pairs requires detailed analysis of the codes that have associated values − which requires knowing which of the tens (or hundreds) of thousands of codes in a terminology have the potential to expose PII. This challenge is illustrated in Fig. 1.

## Proposed Solutions

As multisite, EHR data-driven research becomes more and more common and the data we share get larger in scale, the risk of this issue causing a sizable HIPAA breach will continue to increase. We have little choice but to come together as a community (including stewards of medical terminologies) and share our best ideas for combatting PII leakage through this mechanism. The following represents an initial list of potential solutions that could be implemented alone or in combination to decrease the risk of unintentional PII exposure when using code-value pair EHR data. Each solution poses a unique trade-off between maintenance requirements, sensitivity/specificity, and compute costs.

### Creation and maintenance of “disallow” code lists

It is entirely logical to attempt to compile a complete list of codes to automatically remove from any EHR dataset intended to be deidentified or HIPAA limited. The terminologies included in such a list could be limited to those that can be used in code-value pairs; thus, there is no need to include ICD-10-CM codes, which do not take on a paired value, whereas terminologies like LOINC and SNOMED-CT can. However, our initial analyses of LOINC and SNOMED-CT have revealed the difficulty (if not impossibility) of compiling a truly complete list. One can search terminologies for code descriptors containing, for example, the phrase “Patient Last Name” − but that is not a guarantee that (1) that phrase will capture all synonyms for “last name” in the terminology or (2) a given institution isn't “overloading” an innocuously named code with an unexpected identifier. Moreover, our analysis uncovered codes that may not contain HIPAA identifiers, but still could pair with data that are not desirable to include in a deidentified dataset (e.g., SNOMED 394571004, “Employer”). Due to their wide variability, it is likely impossible to capture all such codes. However, it is certainly possible to compile a list that represents the codes that we and others have been able to find thus far, so long as it is accepted that such a list cannot be considered complete, and that committed maintenance will be necessary in order to stay up to date with changing terminologies. To this end, we have created a table compiled with assistance from LOINC, OHDSI ATHENA [4], and TriNetX. A “live” version of this table that will track updates over time is hosted at https://github.com/data2health/next-gen-data-sharing/blob/master/CodesWithPPIPotential.csv. We welcome additions to this list from the community.

### Creation and maintenance of “allow” code lists

A more drastic measure is to limit the contents of data domains that use code-value pairs to a list of known, allowable codes. This would drastically reduce the number of rows of available laboratory or observation data, but would all guarantee that the values present in the data are known not to contain PII. Using descriptive statistics, one could determine the top, say, 500 laboratory or observation codes present in the raw data and limit the data made available for deidentified or HIPAA limited sets to those codes only. Special requests for codes outside of the default set could be considered on a caseby-case basis and would allow a data broker to manually review the values associated with the newly requested codes for potential PII. Like a disallow list, this allow list would also require maintenance and regular review, particularly as research priorities change. Indeed, maintenance may be even more critical with the allow list method, to avoid unintentionally excluding new concepts and codes as time goes on.

### Special handling for string-formatted values

A compromise option between the disallow and allow lists may recognize that reidentification risk is heightened when a value field is in string format, such as names or addresses. Under this assumption, codes taking integer or floating-point values may be managed by disallow lists to avoid identifiers like telephone numbers, Social Security numbers, and ZIP codes, while fields taking free-text values may only be passed if they appear on an allow list.

### Creation of PII “sniffer” algorithms

In combination with any of the above solutions, targeted regular expressions can be built into ETL processes in order to “sniff” out any additional PII (or potential PII) − such as any data in the format of a phone number, or a person or place name. (E.g., the regular expression "Mr\.|Mrs\.|\bMiss\b|Dr\.|, M\.?D\.?" will find any string with an English name prefix.) Depending on risk tolerance, the expressions could err toward sensitivity or specificity and could be tweaked over time to meet an institution’s needs. (Note, however, that extensive regular expression matching during ETL may add significant processing time and should therefore not be relied upon as a sole solution, but rather an extra protection against edge cases.) Rather than programming a sniffer from scratch, institutions can make use of scripts and methods from the large body of prior work on named entity recognition and clinical text deidentification [5,6].

Other algorithmic rules may also prove useful, such as automatically quarantining records with lengthy string values (which could signal the presence of free text). If these approaches are implemented, records that match the regular expressions or rules can be quarantined in a separate table or staging area to be manually reviewed by a data broker. Thus, in addition to adding another layer of PII protection, another advantage of these approaches is the potential to uncover new problem codes to add to disallow lists going forward.

## Conclusion

As trusted stewards of sensitive data, maintaining institutional and public trust is critical in order to perform the work of clinical informatics. However, the widening scope of structured data in EHRs has made the seemingly straightforward task of dropping or masking identifiers much more complex. Ceasing to share data to prevent inadvertent PII leaks would be extraordinarily detrimental to the growing popularity and scientific potential of multisite, EHR data-driven research. To avoid this outcome, we have proposed a number of solutions that can be implemented immediately on a local level. However, we also see a need for a larger community conversation on this topic, such that these solutions can be made consistent and sustainable over time, and risk can be minimized for our institutions’ patients.
